# Anti-hyperalgesic effects of calcitonin on neuropathic pain interacting with its peripheral receptors

**DOI:** 10.1186/1744-8069-8-42

**Published:** 2012-06-07

**Authors:** Akitoshi Ito, Mineko Takeda, Takeshi Yoshimura, Takayuki Komatsu, Takeshi Ohno, Hiroshi Kuriyama, Akio Matsuda, Megumu Yoshimura

**Affiliations:** 1 Laboratory for Development Pharmacology, Pharmaceuticals Research Center, Asahi Kasei Pharma Co. Ltd, 632-1 Mifuku, Izunokuni-shi, Shizuoka 410-2321, Japan; 2 Laboratory for Pharmacology, Pharmaceuticals Research Center, Asahi Kasei Pharma Co. Ltd, Shizuoka, 410-2321, Japan; 3 Pharmaceuticals Research Center, Asahi Kasei Pharma Co. Ltd, Shizuoka, 410-2321, Japan; 4 Graduate School of Health Sciences, Kumamoto Health Science University, Kumamoto, 861-5598, Japan

**Keywords:** Elcatonin, Calcitonin, Peripheral nerve excitability, Neuropathic pain, CCI model, Na^+^ channel, Analgesia

## Abstract

**Background:**

The polypeptide hormone calcitonin is clinically well known for its ability to relieve neuropathic pain such as spinal canal stenosis, diabetic neuropathy and complex regional pain syndrome. Mechanisms for its analgesic effect, however, remain unclear. Here we investigated the mechanism of anti-hyperalgesic action of calcitonin in a neuropathic pain model in rats.

**Results:**

Subcutaneous injection of elcatonin, a synthetic derivative of eel calcitonin, relieved hyperalgesia induced by chronic constriction injury (CCI). Real-time reverse transcriptase-polymerase chain reaction analysis revealed that the CCI provoked the upregulation of tetrodotoxin (TTX)-sensitive Nav.1.3 mRNA and downregulation of TTX-resistant Nav1.8 and Nav1.9 mRNA on the ipsilateral dorsal root ganglion (DRG), which would consequently increase the excitability of peripheral nerves. These changes were reversed by elcatonin. In addition, the gene expression of the calcitonin receptor and binding site of ^125^I-calcitonin was increased at the constricted peripheral nerve tissue but not at the DRG. The anti-hyperalgesic effect and normalization of sodium channel mRNA by elcatonin was parallel to the change of the calcitonin receptor expression. Elcatonin, however, did not affect the sensitivity of nociception or gene expression of sodium channel, while it suppressed calcitonin receptor mRNA under normal conditions.

**Conclusions:**

These results suggest that the anti-hyperalgesic action of calcitonin on CCI rats could be attributable to the normalization of the sodium channel expression, which might be exerted by an unknown signal produced at the peripheral nerve tissue but not by DRG neurons through the activation of the calcitonin receptor. Calcitonin signals were silent in the normal condition and nerve injury may be one of triggers for conversion of a silent to an active signal.

## Background

Calcitonin is a polypeptide hormone released from the thyroid gland that regulates the calcium homeostasis in vertebrates [1-3] and is used clinically to treat hypercalcemia [4] and osteoporosis [5-7]. In addition, calcitonin has been reported to relieve pain associated with post-menopausal osteoporosis [8], and to ameliorate neuropathic pain associated with lumbar spinal canal stenosis [9], diabetic neuropathy [10], reflex sympathetic dystrophy [11] and post-herpetic neuralgia [12]. Recently, it was also shown that calcitonin inhibits development of complex regional pain syndrome after stroke [13].

Several lines of evidence suggest that the descending serotonergic system is involved in the anti-hyperalgesic effect of calcitonin by modifying the expression of serotonin receptors at the central terminals of primary C afferents in ovariectomy-induced hyperalgesia in rats [14-16]. In contrast to the hyperalgesia associated with post-menopausal states, mechanisms for the anti-hyperalgesic effect of calcitonin on neuropathic pain remain unclear. Moreover, the site of action of the calcitonin effect is still unidentified and there is no report of calcitonin receptor (CTR) expression on DRG neurons or peripheral nerve tissues under normal conditions.

An important role of voltage-gated sodium channels in neuropathic pain states has been established in animal models [17] and several studies exhibit causally linked changes in sodium channel expression and modulation that alters channel gating properties or current density in nociceptor neurons [17]. Biophysical and pharmacological studies identify the sodium channel isoforms Nav1.3, Nav1.7, Nav1.8 and Nav1.9 as particularly important in the pathophysiology of peripheral neuropathic pain [17].

In the present study, we first analyzed the anti-hyperalgesic effects of repeated subcutaneous injections of calcitonin on pain behaviors in chronic constriction injury (CCI)-induced hyperalgesia in rats. Next, we examined a peripheral mechanism for the action of calcitonin. To address this action, the expression of CTR was analyzed on DRG and sciatic nerve tissues. Finally, to confirm that the effect of calcitonin is specific to the neuropathic pain state, the efficacy of calcitonin for the treatment of pain behaviors and sodium channel expression under normal conditions was performed.

## Results

### Anti-hyperalgesic effect of calcitonin on pain behaviors in CCI model rats

**Figure 1:**
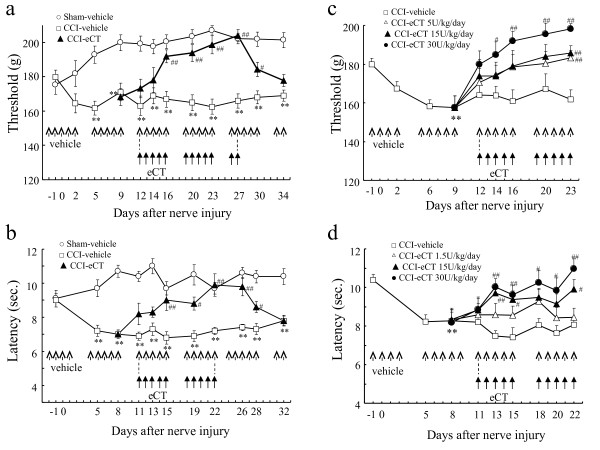
**Anti-hyperalgesic effect of eCT on pain behaviors in CCI rats.** Mechanical hypersensitivity (**a, c**) and thermal hyperalgesia (**b, d**) developed over time on the ipsilateral hind paw in CCI rats. Elcatonin (20 U/kg), given subcutaneously five times a week starting 12 or 11 days after surgery, gradually improved mechanical hypersensitivity (**a**) or thermal hyperalgesia (**b**), respectively. Effects persisted for several days after cessation of the drug. The effects of eCT were dose-dependent on mechanical hypersensitivity (**c**) and thermal hyperalgesia (**d**) in CCI rats. 14 (**a**) or 12 (**b, c, d**) rats were used in each group. All RM-ANOVA revealed the significant differences (P < 0.01). Significant differences by Dunnett’s test or *t*-test: **P < 0.01, as compared to the sham-vehicle or the pre-operation; #P < 0.05, ##P < 0.01, as compared to the CCI-vehicle.

As shown in Figure 1, mechanical hypersensitivity and thermal hyperalgesia developed over time on the ipsilateral hind paw in CCI model rats (Figure 1a,b). Five times a week, elcatonin (eCT; a synthetic derivative of eel calcitonin) (20 U/kg) was given subcutaneously starting 11 or 12 days after surgery. This treatment regimen gradually relieved the mechanical hypersensitivity and thermal hyperalgesia, and these effects persisted for several days after cessation of the drug (Figure 1a,b). The effects of eCT were dose-dependent on both mechanical hypersensitivity and thermal hyperalgesia in CCI model rats (Figure 1c,d).

### Change in Na^+^ channel transcription on ipsilateral L4-L5 DRG at 26 and 27 days after CCI operation

**Figure 2:**
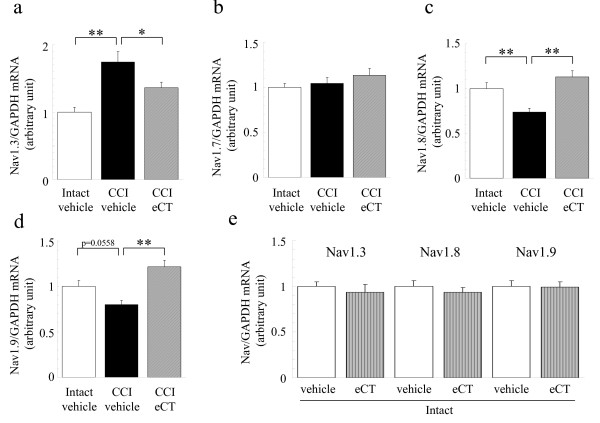
**Change in Na**^**+**^**channel transcription on L4-5 DRG, as measured by real-time RT-PCR.** Elcatonin (15 U/kg), given subcutaneously five times a week from 11 to 26 or 27 days post-surgery, improved the CCI-induced change in Na^+^ channel transcription on ipsilateral L4-5 DRG. Effect of eCT treatment on (**a**) Nav1.3, (b) Nav1.7, (**c**) Nav1.8 and (**d**) Nav 1.9 expression normalized to the endogenous control GAPDH. (**e**) Elcatonin did not affect the gene expression of Na^+^ channels in contralateral intact DRG. The relative amount of target was quantitated by the relative standard curve method (mean in contralateral intact-vehicle group = 1). Each value represents the mean ± SEM(n = 12). ANOVA revealed (**a, c, d**) the significant differences (P < 0.01) and (**b**) no difference (P = 0.3049). Significant differences by Dunnett’s test: **P < 0.01,*P < 0.05, as compared to the CCI-vehicle.

CCI significantly increased the transcription of Nav1.3 but not Nav1.7 on the ipsilateral DRG, which are TTX-sensitive Na^+^ channels, compared to the contralateral intact DRG (Figure 2a,b). In contrast, CCI caused a significant reduction in TTX-resistant Na^+^ channels, Nav1.8 and Nav1.9 mRNA expression on the ipsilateral DRG compared to the contralateral DRG (Figure 2c,d). Subcutaneous administration of eCT (15 U/kg) five times a week from 11 to 27 days post-surgery significantly restored the CCI-induced changes in Nav1.3, Nav1.8 and Nav1.9 transcription (Figure 2a,c,d), but did not affect the expression of Nav1.7 mRNA (Figure 2b). Our results confirmed that sham operation did not affect the gene expression of these Na^+^ channel subtypes in the rat DRG (data not shown). In addition, the same ineffectiveness was observed in contralateral intact DRG (Figure 2e).

### Calcitonin receptor expression on the sciatic nerve tissue and L4-L5 DRG

**Figure 3:**
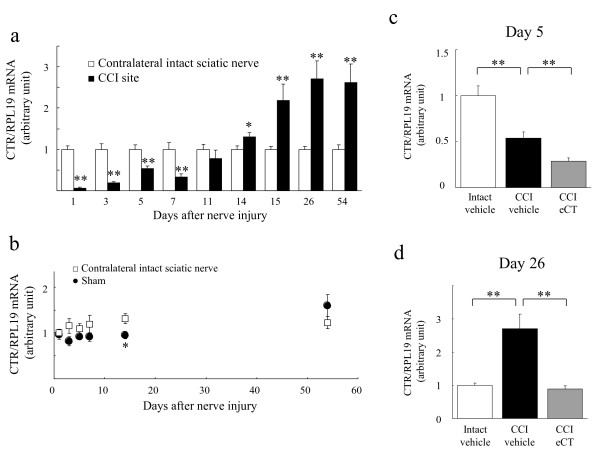
**Changes in expression of calcitonin receptors (CTR) after surgery on the sciatic nerve.** (**a**) The time course of change in CTR mRNA expression in the CCI site. (**b**) The CTR gene expression was slightly influenced in sham operation compared to contralateral sciatic nerve in CCI rats. (**c**) The additional downregulation of CTR mRNA on CCI site by five injections of eCT (20 U/kg/day) immediately following CCI. (**d**) Elcatonin (20 U/kg), injected subcutaneously five times a week from 11 to 26 days post-surgery, suppressed the CCI-induced increase in CTR gene expression to the intact level. CTR gene expression was normalized to the endogenous control RPL19. The relative amount of target was quantitated by the relative standard curve method (mean in intact-vehicle group at day 1 = 1). Each value represents the mean of 6 data points (12 animals) ± SEM. (**a**)(**b**) Two-way or (**c**) one-way ANOVA exhibited the significant differences (P < 0.01). Significant differences by *t*-test or Dunnett’s test: **P < 0.01,*P < 0.05, as compared to the CCI-vehicle.

To elucidate the site of action of calcitonin, we first examined CTR gene expression in sciatic nerve tissue and DRG in sham-operated rats. Real-time reverse transcriptase-polymerase chain reaction (RT-PCR) analysis revealed that there was CTR gene expression in sciatic nerve tissue (Figure 3) but not in the DRG (data not shown). Quantitative study indicated that the amount of CTR mRNA on sciatic nerve tissue was about 1/564 and 1/5 that on the hypothalamus and spinal cord, respectively. Because cells expressing CTR may be damaged and/or proliferating with nerve injury, we examined a change in the expression of CTR in the sciatic nerve after surgery.

The CCI operation induced a change in the time course of CTR mRNA expression (Figure 3a). Immediately after the nerve injury, CTR gene transcription decreased remarkably, recovered gradually by 11 days post-surgery, and then increased after 14 days post-injury (Figure 3a), while the CCI did not induce a change in gene expression of CTR in the side contralateral to the injury (Figure 3b). The sham operation slightly influenced the CTR gene expression (Figure 3b). Immediately following the nerve injury, five times injection of eCT (20 U/kg/day) induced an additional downregulation of CTR mRNA on CCI site (Figure 3c). The CCI-induced increase in CTR gene expression was downregulated to the normal, i.e. intact level by eCT (20 U/kg) injected subcutaneously five times a week from 11 to 26 days post-surgery (Figure 3d).

### Analysis of ^125^I-calcitonin binding site in the sciatic nerve membrane obtained from sham-, CCI- or eCT-treated CCI rats

**Figure 4:**
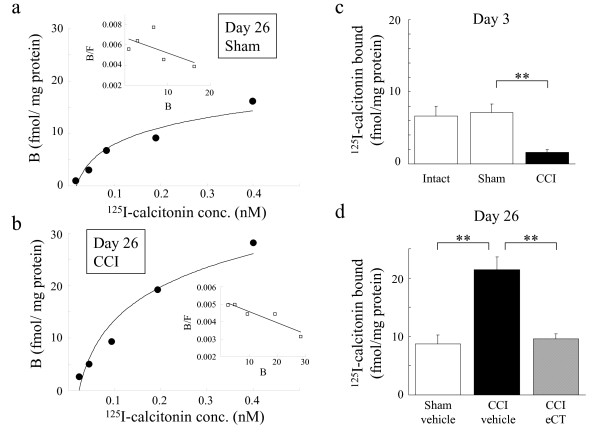
**Analysis of**^**125**^**I-calcitonin binding site in the sciatic nerve membrane.** (**a**) Saturation analysis of ^125^I-CT binding to mixed membranes taken from 15 sciatic nerves in sham rats. (**b**) Saturation analysis of ^125^I-CT binding to mixed membranes taken from 15 sciatic nerves with the CCI regions. Scatchard analysis of specific binding data from (a) or (b) were presented in insert plots, respectively. (**c**) CCI decreased in density of ^125^I-CT (0.2 nM) binding site in the sciatic nerve membrane 3 days after the operation. (**d**) CCI increased the density of ^125^I-CT (0.2 nM) binding sites in the sciatic nerve membrane at 26 days post-surgery. Elcatonin (20 U/kg), injected subcutaneously five times a week from 11 to 26 days post-surgery, decreased in the density of ^125^I-CT binding site on the ipsilateral side to the sham level. (**c**) (**d**): Each value represents the mean of 6 data points from 12 animals ± SEM. One-way ANOVA exhibited the significant differences (P < 0.01). Significant differences by Dunnett’s test: **P < 0.01, as compared to (**c**) the sham or (**d**) the CCI-vehicle.

Analysis of ^125^I-CT binding showed that there was specific binding to membranes taken from the sciatic nerve tissue in sham and the CCI regions at 26 days after the operation, which were saturated with increasing concentrations of ligand (Figure 4a,b). Scatchard analysis of specific binding data presented in Figure 4a and b showed that the binding site was single both in sham and CCI regions. Unexpectedly, ^125^I-CT binding could not be detected in the DRG (data not shown).

CCI significantly decreased the density of ^125^I-CT (0.2 nM) binding site in the membrane obtained from sciatic nerve tissues at 3 days after operation (Figure 4c). This change corresponded to the decrease in gene expression of CTR at the CCI site (Figure 3a). In contrast, CCI increased the density of ^125^I-CT (0.2 nM) binding sites in the sciatic nerve membrane at 26 days post-surgery (Figure 4d). Injections of eCT (20 U/kg) between 11 and 26 days post-surgery drastically decreased the density of ^125^I-CT binding sites in the CCI rats to the sham level (Figure 4d). This alteration also corresponded to changes in CTR transcription 26 days after surgery (Figure 3d).

### Verification of sciatic CCI region as the site of calcitonin-induced anti-hyperalgesic effect

**Figure 5:**
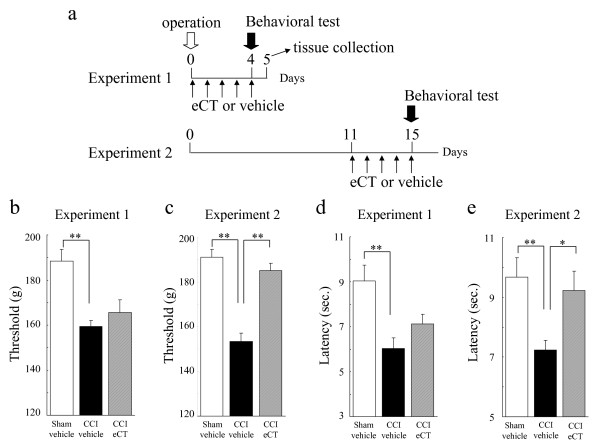
**Verification of the site of action of eCT-induced anti-hyperalgesic effect as a sciatic CCI region.** (**a**) Protocol of each experiment, including operation, behavioral test, eCT injection, and tissue collection. (**b**)(**d**) Reduction of eCT (20 U/kg, s.c.)-induced anti-hyperalgesic effect 4 days after operation (Experiment 1; (**b**) mechanical or (**d**) thermal hyperalgesia, n = 12). (**c**)(**e**) Anti-hyperalgesic effect of eCT (20 U/kg, s.c.) 15 days after operation (Experiment 2; (**c**) mechanical, n = 14 or (**d**) thermal hyperalgesia, n = 12). Each value represents the mean ± SEM. All ANOVA revealed the significant differences (P < 0.01). Significant differences by Dunnett’s test: **P < 0.01,*P < 0.05, as compared to the CCI-vehicle.

**Figure 6:**
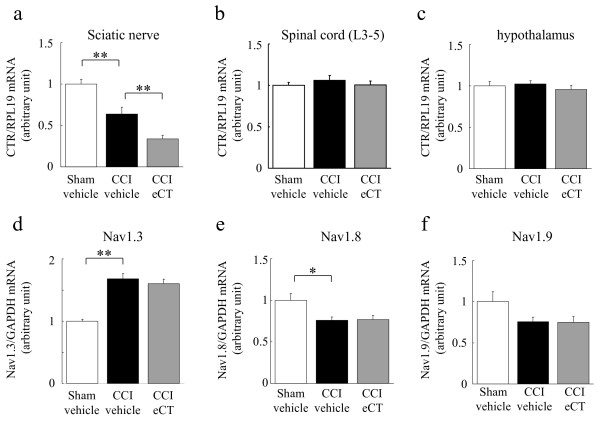
**Changes in mRNA expression of CTR and Na**^**+**^**channels at 5 days after nerve injury.** Changes in CTR mRNA expression on ipsilateral sciatic nerve (**a**), spinal cord (**b**) or hypothalamus (**c**) (Experiment 1, 5 days post-surgery). It was verified that the specific downregulation of CTR mRNA on CCI segments from sciatic nerve in CCI rats and much further reduction in CCI-eCT rats. (**d**)(**e**)(**f**): Disappearance (offset) of eCT-induced normalization for Na^+^ channel gene expression on ipsilateral L4-5 DRGs in CCI rats (Experiment 1, 5 days post-surgery). (d), Nav1.3; (**e**), Nav1.8; (**f**), Nav1.9. Each value represents the mean ± SEM. ANOVA revealed (**a, d, e**) the significant differences (P < 0.01, P < 0.01, P = 0.0113) and (**b, c, f**) no difference (P = 0.6054, 0.6115, 0.0742). Significant differences by Dunnett’s test: **P < 0.01,*P < 0.05, as compared to the CCI-vehicle.

To clarify our assumption that a sciatic CCI region is the site of action of calcitonin, we conducted two behavioral tests together with quantitative RT-PCR for Na^+^ channels and CTR (Figure 5a). In the period of lower expression of CTR at the CCI site (Experiment 1), the eCT (20 U/kg, 5 times injection)-induced anti-hyperalgesic effect was not detected (Figure 5b,d). In fact, CTR mRNAs were decreased on the sciatic CCI segment in Experiment 1 and much further reduced in eCT-treated CCI rats (Figure 6a), but were not changed in the spinal cord and hypothalamus (Figure 6b,c). In contrast, eCT administration from 11 to 15 days after CCI resulted in an anti-hyperalgesic effect (Figure 5c,e, Experiment 2). Furthermore, the eCT-induced normalization of the Na^+^ channel gene expression on the ipsilateral L4-L5 DRG disappeared in Experiment 1 (Figure 6d,e,f).

### Signal and effects of calcitonin under normal conditions

**Figure 7:**
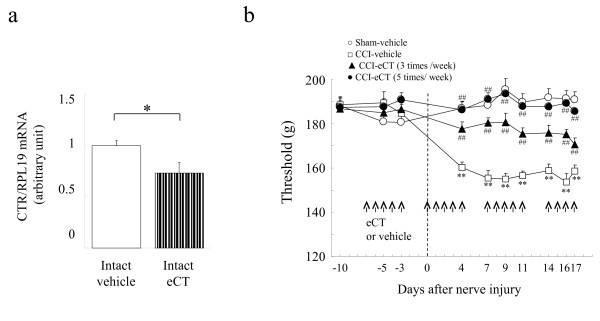
**Influence of eCT on the CTR gene expression and the pain behavior under normal condition.** (**a**) Reduction in CTR mRNA expression on intact sciatic nerve by 5 injections of eCT. (**b**)5 injections of eCT did not affect the pain behaviors under normal conditions before surgery, but prophylactic injections of eCT prevented the CCI-induced hypersensitivity. (**a**) Each value represents the mean of 6 data points from 12 animals ± SEM. Significant differences: *P < 0.05 by *t*-test. (**b**); Each value represents the mean ± SEM. RM-ANOVA showed the significant difference (P < 0.01). Significant differences by Dunnett’s test: **P < 0.01 or ##P < 0.01, as compared to the sham-vehicle or CCI-vehicle, respectively.

As shown in Figure 7a, injections of eCT (20 U/kg/day) induced a downregulation of CTR mRNA on intact sciatic nerves. However, eCT injections did not have an effect on gene expression of Na^+^ channels in intact L4-L5 DRG (Figure 2e). The behavioral study indicated that eCT had no effect under normal conditions before the CCI operation (Figure 7b). Surprisingly, the prophylactic administration of eCT prevented the development of hyperalgesia even though there was little CTR expression (Figure 7b).

## Discussion

We demonstrated that eCT exhibits anti-hyperalgesic effects on neuropathic pain by restoring the CCI-induced abnormal gene expression of Na^+^ channels in the ipsilateral DRG neurons through the activation of CTR. CTR expression was drastically increased by CCI and was confined to the constricted region. Unexpectedly, CTR was expressed in peripheral nerve tissues, including Schwann cells, blood vessels, connective tissue, and others, but not on DRG neurons. Though we detected the more expression of CTR in spinal cord and hypothalamus than peripheral nerve tissue, the anti-hyperalgesic effect and normalization of Na^+^ channel mRNA by eCT was parallel to the change of the CTR mRNA expression in peripheral nerve tissues but not in the spinal cord and hypothalamus. Therefore, our studies suggest that the eCT-induced recovery of the abnormal expression of Na^+^ channel mRNA in DRG neurons could be mediated by a “calcitonin signal” released as a result of the activation of CTR to prevent the action of unknown factor(s) from the injured peripheral tissues. This might contribute to the anti-hyperalgesic effect of calcitonin on neuropathic pain.

**Figure 8:**
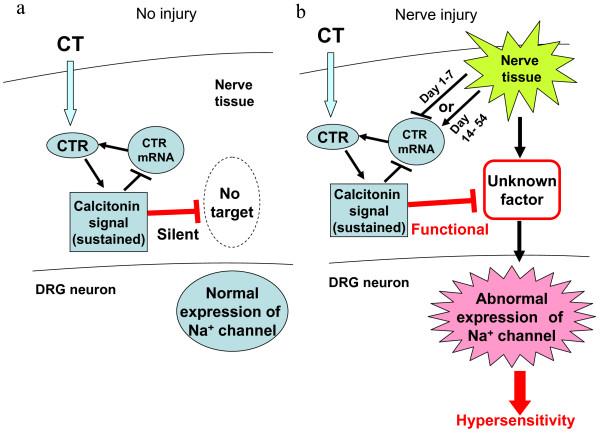
**Possible mechanisms for the inhibition by calcitonin of the abnormal expression of sodium channels.** (**a**) The action of calcitonin under normal conditions. Calcitonin-induced signal via CTR are silent under normal condition. (**b**) Nerve injury not only induces the abnormal expression of Na^+^ channels but also could trigger the activation of the silent signal to a functional one. Calcitonin may exert anti-hyperalgesic effects following the inhibition of the abnormal expression of Na^+^ channels via these functional signals.

The downregulation of CTR expression is well known. It has been reported that the stimulation of osteoclast by calcitonin causes not only an inhibition of bone resorption via activation of protein kinase A [3], but also a decrease in ^125^I-calcitonin binding, which is related to the amount of CTR mRNA [18]. Therefore, the downregulation of CTR mRNA could be mediated by calcitonin itself or an unknown calcitonin-induced signal following the activation of CTR. The downregulation of CTR most likely controls the generation of the calcitonin signal. Our results suggest the existence of a peripheral CTR-mediated system that serves as a feedback mechanism to regulate the levels of calcitonin signal. On the other hand, eCT injections did not influence the CTR mRNA expression in the spinal cord and hypothalamus, because eCT could not pass through the blood brain barrier,

Calcitonin signal may also be induced under the normal conditions by eCT acting on the CTR in peripheral nerve tissues, because eCT suppressed CTR mRNA in the intact nerve tissue (Figure 7a). However, this signal was thought to be non-functional based on the fact that eCT had no influence on the expression of Na^+^ channels (data not shown) or on the behavioral responses before surgery (Figure 7b). As shown our speculation in Figure 8a, the “silent” signaling pathway of CT will be dependent on the unknown factor which induced the abnormal expressions of Na^+^ channel. Therefore, the calcitonin signal was presumed to be silent under normal conditions (Figure 8a); however, a nerve injury could trigger the silent unknown factor to active. Application of eCT activated the calcitonin signal which prevent the activation of unknown factor, resulting in normalization of Na^+^ channel expression (Figure 8b).

A recent review shows that Nav1.3, Nav1.7, Nav1.8 and Nav1.9 play pivotal roles in pain transmission [17]. Nav1.3 expression, a low threshold sodium channel, is upregulated in adult rat DRG neurons by peripheral nerve injury [19,20]. In contrast, the expression of Nav1.8 and Nav1.9, high-threshold TTX-resistant sodium channels, is significantly attenuated in injured neurons [19,21,22]. These observations suggest that the injured nerves become sensitive to small membrane potential changes and could initiate spontaneous spikes. Our results demonstrating the alteration of sodium channel expression following CCI were consistent with previous reports [19-22]. Moreover, repeated administration of eCT normalized the gene expression of Na^+^ channels in CCI rats (Figure 2). This normalization of sodium channel mRNA by eCT parallels the anti-hyperalgesic effect and the change of the CTR mRNA expression in peripheral nerve tissues. Accordingly, the anti-hyperalgesic action of calcitonin in CCI rats could function to normalize the sodium channel expression, which was exerted by a calcitonin signal produced through the CTR in peripheral nerve tissues but not in DRG neurons.

Cellular localization of CTR in peripheral nerve tissues and a factor related to calcitonin signalling has not been identified. We tried to detect a specific band and signal by immunohistochemistry and in situ hybridization, but we could not. The reasons may be that it was unavailable to get a specific anti-CTR antibody or CTR expression was too low to detect under the condition we used. After nerve injury, the induction of demyelination and proliferation of Schwann cells is well known [23,24]. We, therefore, assumed that the CTR was expressed in Schwann cells, and the decrease or increase in the expression of CTR level might contribute to the demyelination or proliferation of Schwann cells. Previous studies have shown that glial cell line-derived neurotrophic factor (GDNF) or nerve growth factor (NGF) regulates the activity of Na^+^ channels or the Na^+^ current density in DRG neurons [25,26]. Schwann cells are known to produce these neurotrophic factors [27,28]. The calcitonin-induced signal, via the activation of CTR in peripheral nerve tissue, could modify those factors and consequently restore the abnormal expression of Na^+^ channels. Cellular distribution of CTR and factors related to calcitonin including GDNF and NGF are under study.

Our previous studies demonstrate that hyperalgesia observed in ovariectomized (OVX) rats, a model for osteoporosis, is attributed to the elimination or reduction of serotonin receptors expressed at the primary C afferent terminals in the spinal cord [14,29]. The change in serotonin receptor expression is restored by repeated injection of eCT [14,29]. Considering the drastic change in the level of expression of serotonin receptors on DRG neurons in OVX rats, calcitonin may produce an anti-hyperalgesic effect via CTR on peripheral nerve tissue.

Although eCT disappeared within 2 h from human [30] and rat plasma (in house data) following an injection, subsequent injection of eCT gradually enhanced the anti-hyperalgesic effect (Figure 1). In addition, the anti-hyperalgesic effect was maintained for several days after cessation of eCT administration (Figure 1). Therefore, the calcitonin system might be sustained for several days and the accumulation of signals probably increases the strength of the anti-hyperalgesic effect.

In spite of the low levels of expression of CTR before operation, the preventive injection of eCT inhibited the development of hyperalgesia (Figure 7). In contrast, we could not detect any anti-hyperalgesic effect when eCT injection was performed at the acute phase after nerve injury (Figure 5b and d). It could be speculated that sustained CT signal before the surgery contributed the prevention of hyperalgesia, while the level of CTR was significantly decreased by nerve injury (Figure 5b and d).

## Conclusions

Our study, for the first time, revealed that there appeared to be a CTR-mediated system which might regulate the excitability of primary afferents by activation of calcitonin-induced signals via the calcitonin receptors to control the sodium channel transcription in DRG neurons. We also showed that this CTR-mediated system was silent under normal conditions but became active following nerve injury, and this system exhibited to provide the negative feedback. The accumulation and maintenance of the calcitonin-induced signal and further analysis of the CTR-mediated system in the peripheral nerve tissue may be one of plausible strategies for alleviate neuropathic pain.

## Methods

### Animals and surgical procedure

All experiments performed were approved by the Institutional Animal Care Committee of the Pharmaceutical Research Center of Asahi Kasei Pharma Corporation and the experimental procedures were conducted in accordance with the Guiding Principles for the Care and Use of Animals recommended by the Physiological Society of Japan.

Male Sprague–Dawley rats (7 weeks-old; 230-370 g) purchased from Charles River laboratory (Atsugi, Japan) were used. The rats were individually housed in a room in which the temperature was controlled to 23 ± 3°C and humidity to 55 ± 10%, with a 12-h light–dark cycle and free access to food and water.

The CCI model rats were made according to the method described by Bennett and Xie [31] with a slight modification. Briefly, under ether anesthesia, the right sciatic nerve was exposed, and 4 loosely constrictive ligatures, using braided silk 4-0 (Niccho Industry Co. Ltd., Tokyo, Japan), were made around the sciatic nerve at the mid-thigh level in an area 5 mm in length. The incision was then closed with braided silk sutures (2-0; Natsume Seisakusho Co. Ltd., Tokyo, Japan). In sham-operated rats, the sciatic nerve was exposed without ligation.

### Drugs and treatments

Elcatonin (Asahi Kasei Pharma Corporation, Tokyo, Japan), a synthetic derivative of eel calcitonin, was dissolved in 0.1 mM sodium acetate buffer (pH 5.5) with 0.9% sodium chloride and 0.02% bovine serum albumin, and administered subcutaneously 5 times per week at a dose of 1.5, 5, 15, 20 or 30 U/kg/day in a volume of 1.0 ml/kg.

### Behavioral analysis

Thermal hyperalgesia was monitored before surgery. Thermal hypersensitivity was tested according to the Hargreaves procedure [32] using the plantar test (Ugo Basile, Varese, Italy). Briefly, animals were placed in a clear Plexiglas box and allowed to acclimatize. A constant intensity radiant heat source was aimed at the midplantar area of the hind paw. The time from initial heat source activation until paw withdrawal was recorded. The cutoff time was set for 22.5 s. Mechanical hyperalgesia was measured using the Randall-Selitto procedure with an analgesic-meter (Ugo Basile, Varese, Italy) which exerts a force (g) that increases at a constant rate. The investigator confirmed the effects of eCT by masking the animal conditions.

### Real-time RT-PCR analysis

Rats were sacrificed by decapitation under ether anesthesia, and the L4-L5 DRG, sciatic nerve, spinal cord and/or hypothalamus were rapidly removed. The tissue samples were immersed in 0.5 mL RNA*later* (Ambion, Austin, TX), and then stored at -80°C until use. RNA was extracted by a single step using TRIzol (Invitrogen, Carlsbad, CA) and chloroform. After centrifugation at 15000 rpm for 15 min, the RNA-containing aqueous phase was precipitated in isopropanol. The RNA pellet was then washed once in 75% ethanol and re-suspended in μL of RNase-free water. Total RNA from each sample was extracted using Qiagen RNeasy mini columns with DNase I (QIAGEN, Tokyo, Japan) to reduce contamination of genomic DNA prior to PCR analysis.

Nav1.8 and Nav1.9 probes and primers were designed as Sleepers [22]. Nav1.3, CTR (both C1a and C1b) and RPL19 probes and primer were designed by using Primer Express (Applied Biosystems, Foster City, CA). BLAST searches were performed to avoid sequence homology with other genes. Commercially available pre-developed TaqMan reagents (Applied Biosystems) were used for Nav.1.7 (Rn00591020_m1) and Rodent GAPDH Control Reagents (VIC Probe, 4308313). GAPDH or RPL19 was used as an endogenous internal control to normalize. Target genes were amplified by using specific primers for Nav1.8 (forward: 5^′^-TGGTCAACTGCGTGTGCAT-3^′^; reverse: 5^′^-AATCAGAGCCTCGAAGGTGTAAA-3^′^; probe: 5^′^-FAM-CCGAACTGATCTTCCAGAGAAAGTCGAGTACGT- TAMRA-3^′^), Nav1.9 (forward: 5^′^-TGCCCTACCCACCTCACAAC-3^′^; reverse: 5′-CCGGGCTAGTGAGCTGCTT-3^′^; probe: 5^′^-FAM-TICAGGCCGGTGACCTCCCTCC-TAMRA-3^′^), Nav1.3 (forward: 5^′^-CCAATAACACGGGCATCGA-3^′^; reverse: 5^′^-CACC^′^CCGCTGGTGGTT-3^′^; probe: 5^′^-FAM-ATAAGCAAAGAGCTTAACTACCTT-3^′^ (TaqMan MGB)), CTR (forward: 5^′^-GCCCTGACTACTTTCCGGACTT-3^′^; reverse: 5^′^-GGTGTCTAAACCACTCTCCATTTTC-3^′^; probe: 5^′^-FAM-ACCCAACAGAAAAGGTTTCAAAATACTGCGA-TAMRA-3^′^), RPL19 (forward: 5^′^-GACCCCAATGAAACCAACGA-3^′^; Reverse: 5^′^-TCAGGCCATCTTTGATCAGCTT-3^′^; Probe: 5^′^-FAM-CGCCAATGCCAACTCTCGTCAACAG-TAMRA-3^′^).

Primers for GAPDH and the others were used at a final concentration of 100 and 900 nM, respectively, whereas the probes were used at a final concentration of 200 and 250 nM, respectively. Real-time RT-PCR was performed with the TaqMan Onestep RT-PCR reaction mix Reagent (Applied Biosystems). Amplification was done in a 50-μl final volume under the following cycling conditions: 30 min at 48°C, 10 min at 95°C and then 40 cycles of 95°C, for 15 s each, followed by 60°C for 1 min. To determine levels of transcripts, the relative standard curve method [33,34] was used. Standard curves were constructed using serial dilutions of RNA from each tissue. Standards and experimental conditions were amplified in duplicate [Additional file 1, 2, 3 and 4.

### ^125^I-calcitonin binding assay

Rats were sacrificed by decapitation under ether anesthesia, and the DRG and sciatic nerve were rapidly removed. The respective tissues from two rats were pooled, homogenized in 4 ml of ice-cooled 10% sucrose, and centrifuged at 1000×*g* for 10 min. The supernatant was removed and further centrifuged at 31000×*g* for 20 min. The pellet was homogenized in 4 ml of ice-cold 50 mM Tris–HCl, pH 7.4, and centrifuged at 31000×*g* for 20 min. The pellet was homogenized in the same buffer. The suspension was then centrifuged as above, and the final pellet was resuspended in the same buffer and stored at -80°C until use. The membrane suspensions were melted rapidly and added to ice-cold binding buffer (50 mM Tris–HCl, pH 7.4, 1 mM EDTA) with 20 mg/ml bovine serum albumin. Each of the membrane solutions was incubated in triplicate with 0.025, 0.05, 0.1, 0.2 or 0.4 nM ^125^I-calcitonin salmon (Peninsula Laboratories, San Carlos, CA) at 25°C for 60 min (0.5 ml of total volume per tube). Nonspecific binding was defined with 2 μM unlabeled eCT. The binding reaction was terminated by rapid filtration under vacuum through 0.3% polyethyleneimine presoaked GF/C filters. The filters were washed four times with 3.5 ml of the binding buffer. Radioactivity was measured using a gamma counter COBRA II (PerkinElmer, Waltham, MA). Protein concentration was determined using a BCA protein assay kit (PIERCE, Rockford, IL).

### Statistical analysis

All results are presented as mean ± SEM. Differences were considered statistically significant when p < 0.05. Effects of eCT in behavioral tests (Figure 1a,b,c,d and 7) were analyzed using two-way repeated measure analysis of variance (RM-ANOVA). CCI-induced hyperalgesia were done by one-way (Figure 1c and 1d) or two-way (Figure 1a,b and 7) RM-ANOVA. Other multiple groups’ data were analyzed by one-way or two-way ANOVA. Multiple and two group’s comparisons were done using post hoc Dunnett’s test and *t*-test, respectively. The calculation was done using SAS software Version 8.2 (SAS Institute Japan Ltd., Tokyo, Japan).

## Abbreviations

CCI: Chronic constriction injury; TTX: Tetrodotoxin; DRG: Dorsal root ganglion; CTR: Calcitonin receptor; eCT: Elcatonin; RT-PCR: Reverse transcriptase-polymerase chain reaction; OVX: Ovariectomized.

## Competing interests

AI, MT, TY, TK, TO, HK and AM are employees of Asahi Kasei Pharma, the manufacturer of elcatonin. The author declare that MY have no competing interests.

## Authors' contributions

AI conceived of the study, performed the design of the study, carried out the collection of sciatic nerve tissues and DRG, the RT-PCR analysis, the binding assay, and the data analysis, and drafted the manuscript. MT carried out the CCI surgery and the behavioral tests. TY performed the collection of sciatic nerve tissues and hypothalamus, and helped the CCI surgery and the drug injection. TK performed the collection of spinal cord, and helped the CCI surgery. TO participated in the RT-PCR analysis, helped the design of the study. HK and AM participated in the design and coordination of the study. MY performed the design of the study, and contributed critically revising the draft of the manuscript. All authors read and approved the final manuscript.

## Supplementary Material

Additional file 1 A raw chart of real time RT-PCR (Nav1.8 mRNA on L4-5 DRG). Click here for file

Additional file 2 **A standard curve of Nav1.8 mRNA on L4-5 DRG, as determined by a raw chart (Additional ****file**** 1).** Click here for file

Additional file 3 A raw chart of real time RT-PCR (GAPDH mRNA on L4-5 DRG). Click here for file

Additional file 4 **A standard curve of GAPDH mRNA on L4-5 DRG, as determined by a raw chart (Additional ****file**** 3).** Click here for file
