# Supplementary figures and images for: Anti-hyperalgesic effects of calcitonin on neuropathic pain interacting with its peripheral receptors
Source: Mol Pain. 2012 Jun 7;8:42. doi: 10.1186/1744-8069-8-42 (PMC3517395; doi:10.1186/1744-8069-8-42)

### Standard Curve

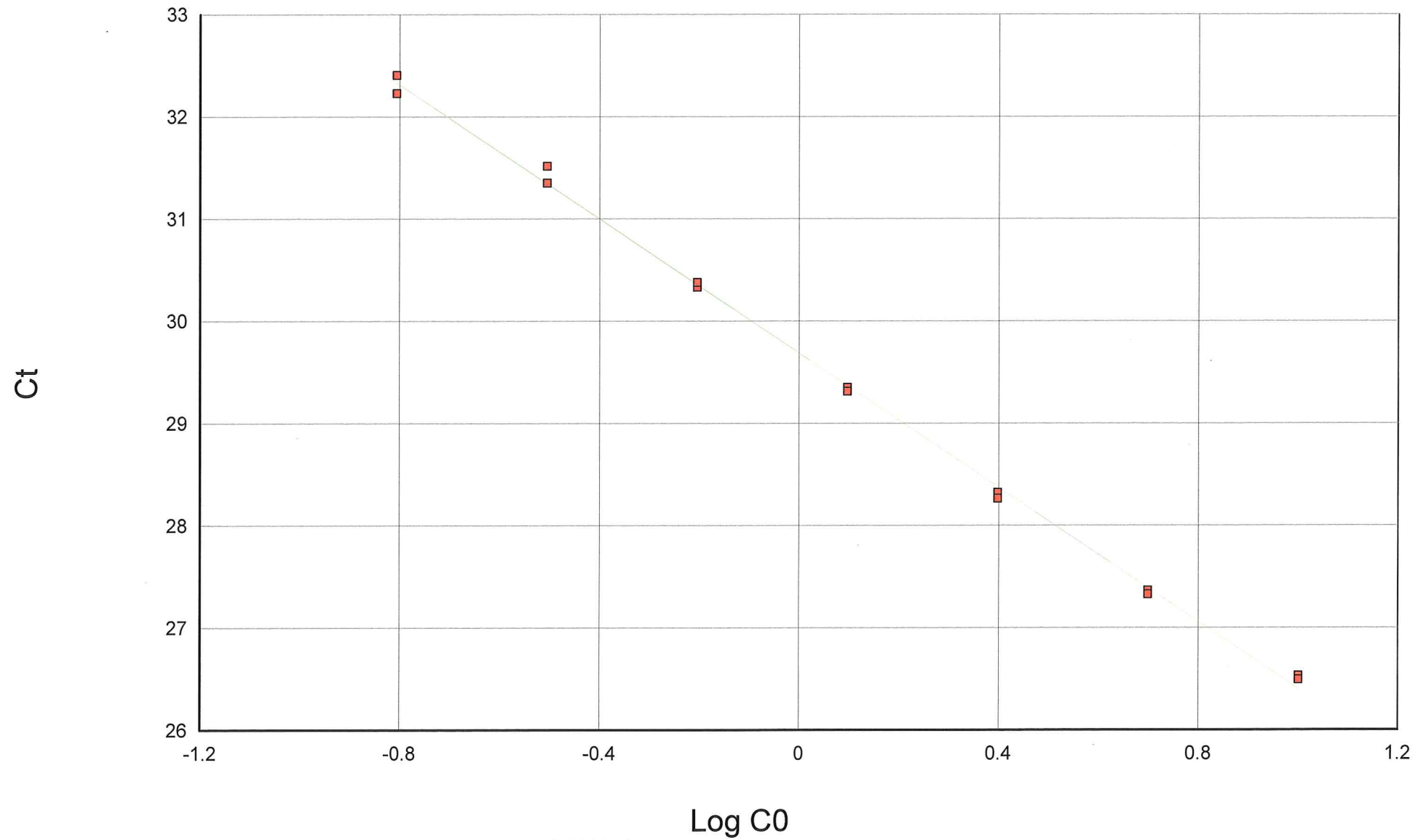

Detector: SNS, Slope: -3.281060, Intercept: 29.687355, R2: 0.998253  
Document: 040507SNS (Absolute Quantification)

Supplement: Additional file 2 — A standard curve of Nav1.8 mRNA on L4-5 DRG, as determined by a raw chart (Additional file 1). [file 1744-8069-8-42-S2.pdf]

### Standard Curve

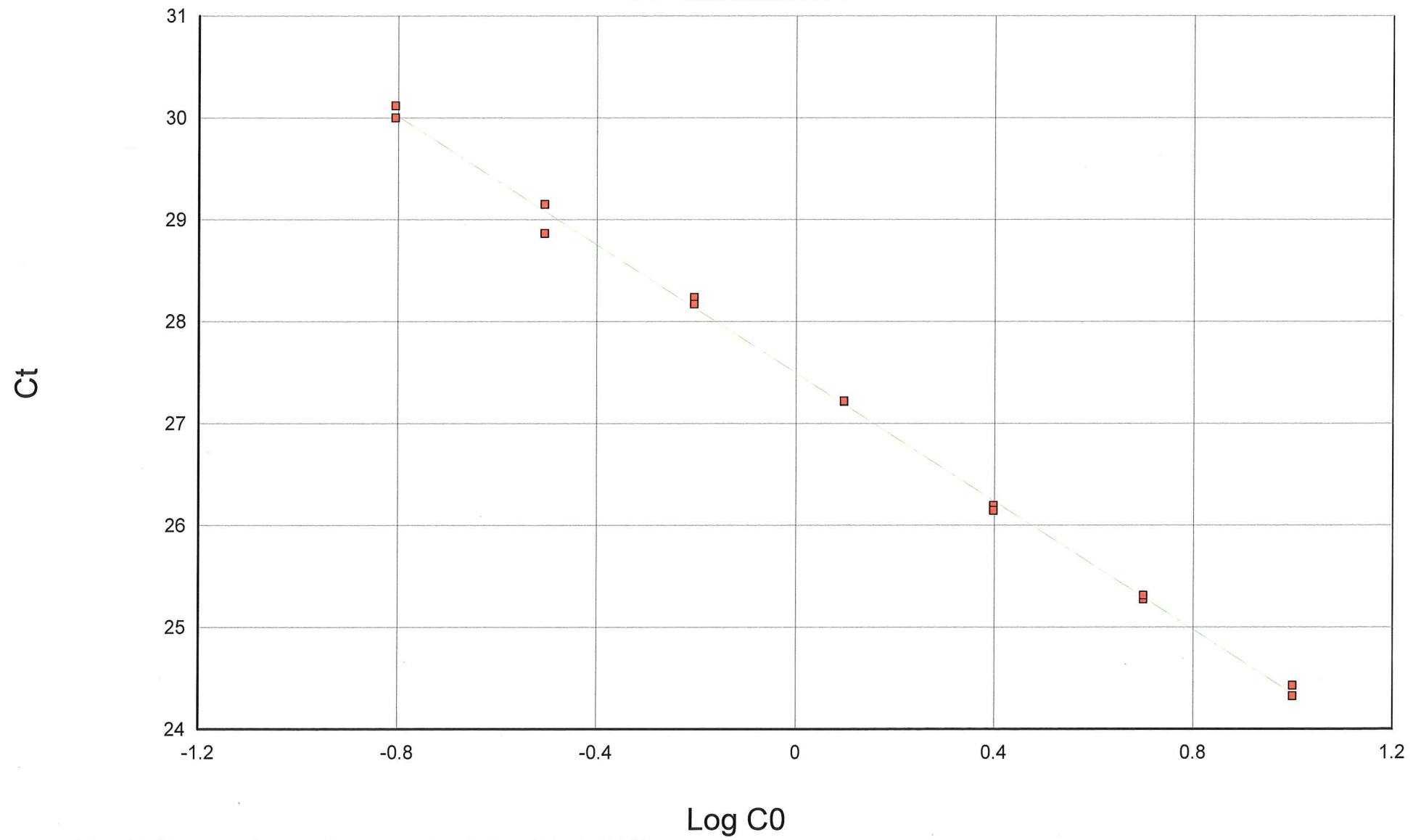

Detector: GAPDH, Slope: -3.145884, Intercept: 27.494377, R2: 0.998165  
Document: 040506gapdh (Absolute Quantification)

Supplement: Additional file 4 — A standard curve of GAPDH mRNA on L4-5 DRG, as determined by a raw chart (Additional file 3). [file 1744-8069-8-42-S4.pdf]
