# Supplementary material for: Anti-hyperalgesic effects of calcitonin on neuropathic pain interacting with its peripheral receptors
Source: Mol Pain. 2012 Jun 7;8:42. doi: 10.1186/1744-8069-8-42 (PMC3517395; doi:10.1186/1744-8069-8-42)
Supplement: Additional file 3 — A raw chart of real time RT-PCR (GAPDH mRNA on L4-5 DRG). [file 1744-8069-8-42-S3.pdf]

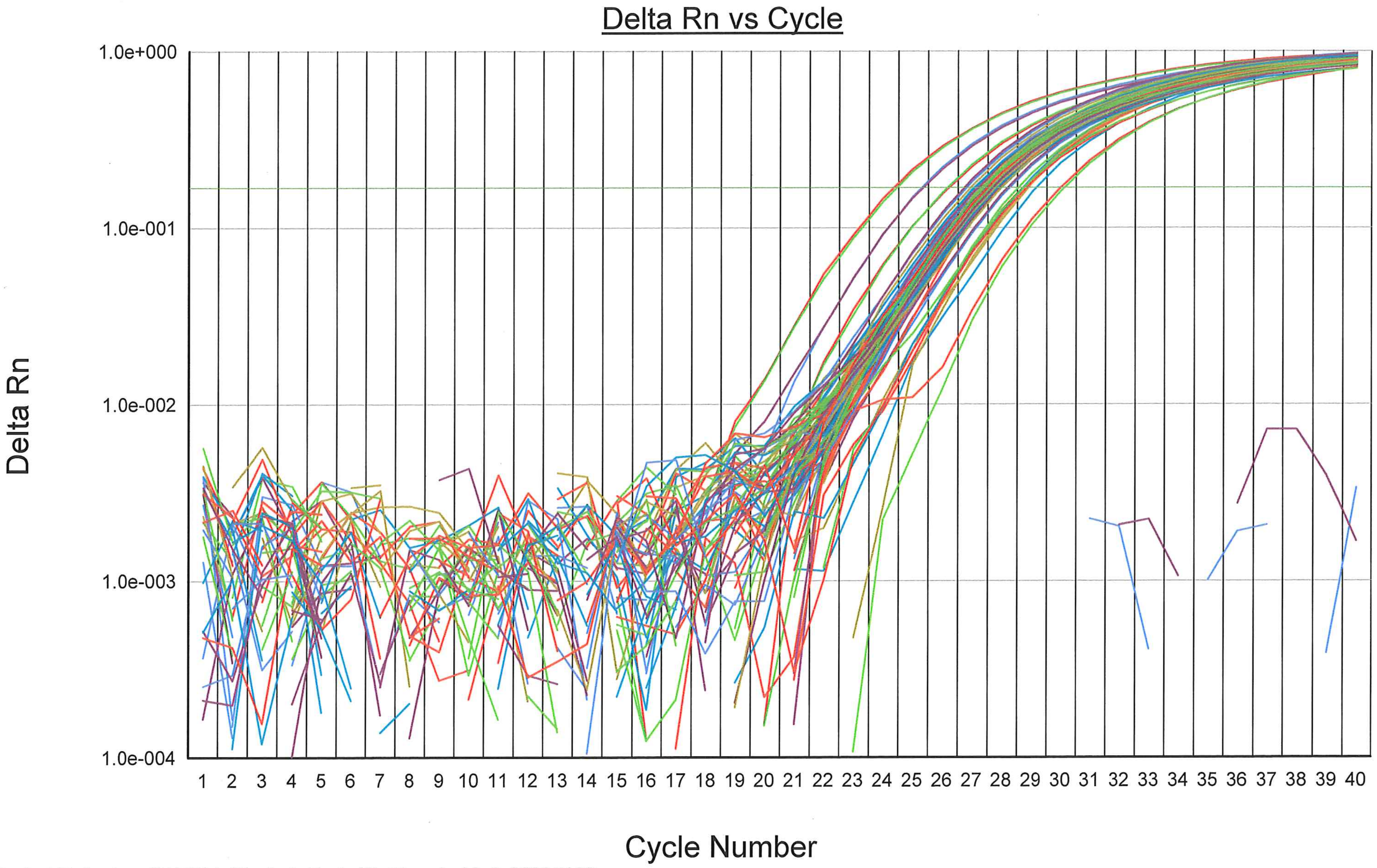

Selected Detector: GAPDH; Start: 1; End: 16; Threshold: 0.16885100
Well(s): A1-H12
Document: 040506gapdh (Absolute Quantification)
